# Influence of Heat Treatment Prior to Fortification on Goitrogenic Compounds, Iodine Stability and Antioxidant Activity in Cauliflower

**DOI:** 10.3390/foods15020315

**Published:** 2026-01-15

**Authors:** Agata Jankowska, Monika Przeor, Katarzyna Waszkowiak, Krystyna Szymandera-Buszka

**Affiliations:** Department of Gastronomy Science and Functional Foods, Faculty of Food Science and Nutrition, Poznań University of Life Sciences, Wojska Polskiego 31, 61-624 Poznań, Poland; monika.przeor@up.poznan.pl (M.P.);

**Keywords:** iodine fortification, cauliflower, pre-treatment, phenolic compounds, goitrogens, antioxidant activity

## Abstract

Iodine deficiency remains a global public health concern. Preliminary studies confirmed that cauliflower can serve as a carrier for iodine salts. However, the influence of its endogenous goitrogenic compounds (phenolic compounds and glucosinolates) on iodine utilisation is not fully understood. This study aimed to assess the potential for enhancing cauliflower’s effectiveness as an iodine carrier through various thermal pre-treatment methods, and to examine how these methods, along with the plant’s endogenous goitrogens, affect iodine stability. Cauliflower was cooked by steaming or boiling (covered or uncovered) and fortified with KI or KIO_3_. Iodine content, selected phenolic compounds (sinigrin, progoitrin, glucobrassicin, gluconapin, indole-3-carbinol) and antioxidant activity (ABTS^●+^, DPPH^●^) were analysed immediately after fortification and after 90 days of storage at 4, 21, or 40 °C under controlled humidity and darkness. The results showed that both the heat-treatment method and storage temperature significantly affected iodine retention and were associated with changes in goitrogenic compounds and antioxidant capacity. Cauliflower demonstrated favourable stability as a carrier of iodine, although phytochemical composition influenced fortification outcomes. These findings suggest that the initial heat treatment of cauliflower significantly affects its effectiveness as a matrix for iodine fortification, likely due to differences in the content of goitrogenic compounds.

## 1. Introduction

Iodine is essential for the synthesis of thyroid hormones, and its deficiency can lead to serious health problems at all stages of human development. Iodine deficiency disorders (IDDs) include hypothyroidism, goitre, impaired cognitive function, delayed physical development, infertility, and subfertility, as well as an increased risk of miscarriage or stillbirth, and congenital disabilities in the foetus [1,2,3]. One of the major risk factors for iodine deficiency is geographical location, due to the uneven distribution of this element in the environment. The largest amounts of iodine are recorded in coastal areas, and the smallest in mountain and post-glacial areas [4,5]. Consequently, iodine deficiency remains a public health concern worldwide, prompting iodine prophylaxis programmes in over 145 countries [6].

Given growing concerns about the adverse health effects of excessive salt consumption, there is a pressing need to identify alternative, healthier carriers of dietary iodine salts [7,8]. Vegetables are a promising option, not only because of their high nutritional value but also because their consumption aligns with various dietary patterns, including vegetarian and vegan diets [9,10]. This is particularly relevant since plant-based diets often exclude major natural dietary sources of iodine such as fish, seafood, dairy products, and meat [11,12,13]. Recent research has confirmed that individuals adhering to vegan diets exhibit significantly lower urinary iodine concentrations compared with other dietary groups, placing them at increased risk of iodine deficiency [14,15]. However, naturally occurring iodine levels in fruits and vegetables are low, as iodine is not essential for plant development, and its concentration in plant tissues strongly depends on soil iodine content [16].

Direct fortification of foods with iodine is associated with several technological challenges. Iodine is an element that is highly sensitive to exposure to light and oxygen, elevated temperatures, and humidity, which may result in losses during processing and storage [17,18]. In addition, iodine fortificants commonly used in food technology exhibit significant oxidative potential: KI acts as a strong reducing agent, while KIO_3_ functions as a strong oxidising agent [19,20]. As a result, these compounds may interact with other food constituents, such as proteins or phenolic compounds, potentially affecting both iodine stability and the phytochemical profile of the fortified matrix [21,22]. Previous studies conducted on vegetable matrices have also shown that the antioxidant activity of the matrix can be altered following the addition of iodine compounds at elevated concentrations [19,20]. Moreover, thermal processing of the raw material has the potential to modify the content of endogenous compounds, suggesting that it may contribute to differences in iodine behaviour in fortified vegetable matrices [23,24].

Cauliflower (*Brassica oleracea* L. var. *botrytis*) is a vegetable that belongs to the Brassicaceae family [25] and widely consumed in many countries and, in particular, by individuals following vegan diets [13]. This makes it a promising candidate as an iodine carrier for populations at risk of deficiency. It is worth taking into account that plants from the Brassicaceae family, including cauliflower, contain bioactive compounds known as goitrogens, which may interfere with thyroid hormone synthesis and exacerbate the consequences of iodine deficiency [26]. Among the phenolic compounds contained in cauliflower, the most important are ferulic acid, chlorogenic acid, gallic acid, catechin and also hydroxycinnamic acids [27], while its major glucosinolates include glucobrassicin, progoitrin, glucoraphanin, sinigrin, gluconapin, glucoiberverin, glucobrassicin, and gluconasturtiin [28]. The adverse effects of certain flavonoids on thyroid function stem from their capacity to inhibit the activity of thyroid peroxidase (TPO), an enzyme essential for thyroid hormonogenesis. In the case of glucosinolates, their mechanism of action may involve either the inhibition of TPO activity or the blockage of the iodide binding site within the sodium/iodide symporter (NIS) [29]. Importantly, recent studies emphasise that the biological effects of goitrogens in humans are complex and strongly dependent on dietary context, processing, and iodine status [30,31,32,33].

Despite the presence of goitrogenic compounds, cauliflower is nutritionally valuable, providing vitamins B1, B2, B3, B5, B6, folic acid, and C, E, and K, as well as omega-3 fatty acids, dietary fibre, potassium, phosphorus, magnesium, manganese, and iron. Moreover, it is a source of numerous bioactive compounds with antioxidant and potential anticancer properties, which support its use as a raw material for functional food production [34,35,36,37]. Recent studies have demonstrated that cauliflower can also serve as a technologically stable matrix for iodine introduced via direct fortification. Optimisation of impregnation parameters has shown that iodine can be efficiently incorporated into vegetable matrices with high retention following freeze-drying, provided that appropriate conditions are applied, including iodine concentration, solution-to-matrix ratio and conditioning temperature. Furthermore, iodine introduced into cauliflower using this approach was shown to remain stable during storage under controlled conditions, supporting the feasibility of this vegetable as a carrier for iodine salts in functional food applications [19,38]. These findings provide a methodological foundation for further investigation while also indicating that iodine stability may be influenced by matrix-specific factors, including processing conditions and the presence of endogenous bioactive compounds.

Processing conditions substantially affect the bioavailability of iodine and the stability of goitrogenic compounds, yet the influence of thermal treatment on these components in iodine-fortified vegetables remains insufficiently understood. Addressing these gaps is crucial for evaluating the safety and feasibility of using cauliflower as an iodine carrier in functional foods. Accordingly, this study focused primarily on the effects of thermal pre-treatment on the stability and retention of iodine in cauliflower, with storage temperature considered as a factor to monitor subsequent changes. It was hypothesised that the type of heat treatment applied to the vegetable matrix influences the recovery of iodine after fortification, its stability during storage, and the content of selected phenolic compounds.

## 2. Materials and Methods

### 2.1. Materials

#### 2.1.1. Iodine Matrix

Cauliflower (*Brassica oleracea* var. *Botrytis* L.) was selected as a matrix for the iodine. The product in a ripe state was purchased in the retail trade in August and September. The KI and KIO_3_ constituted the sources of iodine (Merck, Darmstadt, Germany).

#### 2.1.2. HPLC

Acetic acid, methanol and acetonitrile were supplied by Merck. A Milli-Q water purification system (Millipore, Bedford, MA, USA) was employed to produce distilled water with a resistivity of 18.2 MΩ. A Vortex MX-S (Chem-Land, Stargard, Poland) was used as a vortex mixer.

### 2.2. Methods

#### 2.2.1. Preparation Conditions

The cauliflower was washed under running tap water. The cauliflower was cut into florets. Next, the vegetable was prepared in three variants:

- cooking in boiling water covered (100 °C; 15 min)—BWC,
- cooking in boiling water uncovered (100 °C; 15 min)—BWU,
- steamed (100 °C; 100% steam/10 min) in a convection oven (Rational, Landsberg am Lech, Germany)—CO.

Distilled water was used for heat treatment of cauliflower so as not to accidentally introduce iodine ions with tap water.

#### 2.2.2. Impregnation Conditions

Cauliflower samples, previously cooked using the established methods, were drained and subsequently subjected to homogenization (homogenizer—Foss, Hilleroed, Denmark) to obtain a particle size of 250 μm. This degree of comminution was applied to ensure uniform and effective incorporation of KI or KIO_3_ solutions into the cauliflower matrix. The conditioning step was performed according to the methodology previously described by Zaremba et al. (2022) [38]. The cauliflower samples were conditioned in aqueous KI or KIO_3_ solutions at a solution-to-matrix ratio of 1:1, at −76 °C for 12 h. The applied iodine concentration corresponded to: 2.3 mg/100 g of iodine, achieved using 3.01 mg/100 g of KI or 3.88 mg/100 g of KIO_3_.

The freeze-drying conditions and procedure were described in detail by Zaremba et al. (2022) [38], with the drying time of 28–30 h. After freeze-drying, the vegetables were homogenised (homogeniser—Foss, Hilleroed, Denmark) to obtain a powder particle size of approximately 250 μm, which was subsequently used for further analyses.

#### 2.2.3. Storage Conditions of Fortified Vegetables

The impregnated and freeze-dried cauliflower samples were stored in sealed black glass containers (screw-cap jars; diameter 7 cm, height 10 cm). The influence of storage conditions on the stability of KI and KIO_3_ was tested during storage at 4 ± 1 °C, 21 ± 1 °C or 40 ± 1 °C and 60% of humidity. Changes in iodine content and phenolic compound levels were assessed at predetermined time points during storage, namely after 1, 30, 60, and 90 days.

#### 2.2.4. Stability of Iodine

To assess the iodine stability under different storage conditions, the iodine content of the vegetables was analysed both after iodine application and following storage. Immediately after drying of fortified samples, quantitative changes in the total and inorganic iodine were determined using a macrochemical method with potassium thiocyanate, as described by Kuhne, Wirth, and Wagner [39] and by Moxon and Dixon [40]. Detailed methodological procedures were reported previously by Waszkowiak and Szymandera-Buszka [41].

Iodine content was recalculated and expressed per dry weight. For this purpose, the dry mass (DM) of iodine carriers was estimated by drying at 105 °C to constant weight [42].

#### 2.2.5. Sample Preparation for HPLC

500 mg of each variant of dried vegetables was weighed into Falcon tubes. Then, 16 mL of the extraction mixture (80:20 (*v*/*v*) methanol/H_2_O) was added to the samples. The samples were vortexed to obtain a homogeneous mixture and next sonicated for 30 min at 25 °C in sonicator Elmasonic Med 30 (Elma Schmidbauer GmbH, Singen, Germany). In order to obtain the supernatant, the samples were centrifuged (4000 rpm, 15 min) at centrifuge Heraeus Megafuge 40P (Thermofisher, Osterode am Harz, Germany), and then the supernatant was collected [43]. Then, in order to evaporate the extraction mixture, the samples were placed on a vacuum evaporator Rotavapor R-215 (BUCHI Labortechnik AG, Flawil, Switzerland). This allowed the methanol to evaporate, and the residual water was evaporated on the thermoblock TB-951U (JW Electronic, Warsaw, Poland). The material thus obtained was dissolved in 100% methanol and subjected to centrifugation. Finally, the supernatant was filtered with a polytetrafluoroethylene (PTFE) syringe filter (0.2 μm pore size) and stored at −20 °C until analysed [43].

#### 2.2.6. HPLC Analysis

Phenolic compounds: sinigrin (SG), progoitrin (PG), glucobrassicin (GB), gluconapin (GN), and indole-3-carbinol (IK) contents were determined using HPLC Agilent 1290 Infinity series rapid resolution LC system (Agilent Technologies, Waldbronn, Germany) equipped with a binary pump and autosampler.

Separation was carried out with a Luna Omega 3 um C18 100 ALC Column (150 mm × 4.6 mm, 3.0 μm). Gradient elution was performed using two mobile phases: acidified water containing 0.5% (*v*/*v*) acetic acid (phase A) and acetonitrile (phase B) following a gradient methods described previously [43]: 0–20 min, linear gradient from 0% B to 20% B; 20–30 min from 20% B to 30% B; and 30–35 min from 30% B to 50% B, 35–45 min from 50% B to100% B. The system was then returned to the initial conditions for 10 min. The flow rate was set at 1.0 mL/min. The column temperature was maintained at 25 °C ± 0.8, and the injection volume was 10 μL. UV detection was carried out using DAD set at λ = 240 nm and λ = 280 nm. Samples were analyzed in duplicates. Standard curves were as follows: sinigrin (SG) y = 710.5x − 46.913, progoitrin (PG) y = 400.2x − 304.53, glucobrassicin (GB) y = 303.97x + 213.65, gluconapin (GN) y = 964.68x + 810.5, indole-3-carbinol (IK) y = 921.95x + 34.478.

#### 2.2.7. Antioxidant Activity

Ethanol extracts were prepared by macerating the dried vegetable samples (10 g) with 100 mL of 80% ethanol for 120 min at 21 °C on an incubated shaker (SIF6000R, Jeio Tech (Lab Companion), Yuseong-gu, Daejeon, Republic of Korea), with the extraction vessels protected from light using aluminium foil.

The antioxidant activity was examined based on the free radical-scavenging indices (the DPPH-scavenging capacity [44,45] and the ABTS-scavenging capability [46]). The results were expressed as mg Trolox 100 g^−1^ dry matter of extract [44,45,46].

### 2.3. Statistical Analysis

Statistical analyses were performed using STATISTICA PL 13.3 (Tibco Software Inc., Palo Alto, CA, USA). Differences between mean values were assessed by analysis of variance (ANOVA), followed by Tukey’s post hoc test, with statistical significance accepted at *p* < 0.05. The iodine content of the tested samples was analysed in 6 samples (2 independent samples and 3 measurements for each sample). Hypothesis testing was conducted at a significance level of α = 0.01.

The kinetics of iodine loss during storage were described using the T_25%_ value. defined as the time required for a 25% reduction in the initial iodine content. These values were calculated from an exponential decay mode [47]. Model performance was evaluated based on the coefficient of determination (R^2^) and root mean square error (RMSE). The significance level for all analyses was set at 5%. Relationships between variables were examined using Pearson’s correlation coefficients (*r*), which were interpreted as follows: *r* < 0.200, no linear relationship; 0.200 ≤ *r* < 0.400, linear dependence weak; 0.400 ≤ *r* < 0.700, linear relationship moderate; 0.700 ≤ *r* < 0.900, linear relationship significant; and *r* < 0.900, linear relationship very strong (*p* ≤ 0.05).

## 3. Results

### 3.1. The Influence of Cauliflower Heat Treatment Conditions on Iodine Retention and Storage Stability

The study indicated that cauliflower can serve as a suitable matrix for iodine fortification under the applied experimental conditions, regardless of the iodine form (KI, KIO_3_) or heat-treatment method. Immediately after drying, iodine retention remained high across all treatments (86–96%), with KIO_3_ consistently providing significantly higher retention than KI (*p* < 0.05). Among preparation methods, boiling without cover (BWU) produced the highest post-drying iodine levels for both iodine forms, whereas steaming in a convection oven (CO) resulted in the lowest values (Table 1).

**Figure 1:**
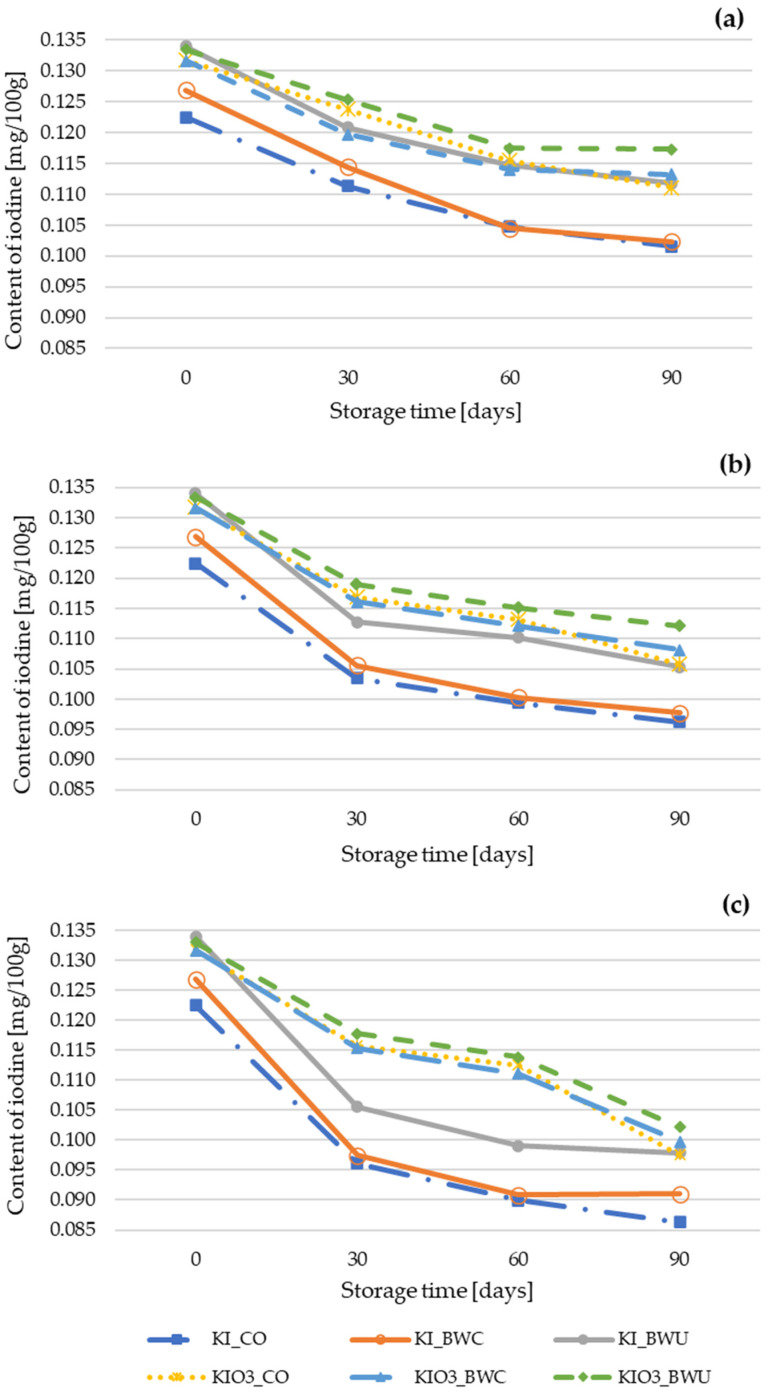
Changes in iodine content in fortified cauliflower during storage in different temperatures (4 °C (**a**), 21 °C (**b**), 40 °C (**c**)) taking into account the iodine form (KI, KIO_3_) and the method of heat treatment (BWC—cooking in boiling water covered, BWU—cooking in boiling water uncovered, CO—steamed in a convection oven).

Across all storage temperatures (4 °C, 21 °C, 40 °C), iodine content decreased progressively over time (0 to 90 days), with the magnitude of losses strongly dependent on temperature and iodine form (Figure 1). The decline was most pronounced at 40 °C, moderate at 21 °C, and least severe at 4 °C, confirming the temperature-dependent instability of iodine in the dried matrix.

After 90 days of storage, KI-fortified samples showed substantially lower retention (70–83%) compared to those fortified with KIO_3_ (74–88%), regardless of treatment method or temperature (Table 1). At each temperature, statistically significant differences (*p* < 0.05) were observed between CO, BWC and BWU, although these method-related effects were smaller than those associated with the form of iodine or temperature.

At 4 °C, the highest retention for both KI and KIO_3_ was observed in BWU, followed closely by CO, while BWC consistently showed the lowest retention. A similar pattern was observed at 21 °C and 40 °C, although differences among methods became less pronounced at elevated temperatures. These observations indicate that preparation-related effects are most relevant under mild storage conditions, whereas high temperature becomes the dominant factor governing iodine degradation.

The kinetic analysis further supported the trends observed in the direct measurements. The estimated T_25_% values (Appendix A Table A1)—the time required for iodine to decrease by 25%—confirmed the superior stability of KIO_3_ relative to KI under all conditions. At 4 °C, T_25_% for KIO_3_ ranged from 150 to 188 days depending on preparation method, whereas KI exhibited markedly shorter T_25_% values (97–120 days). This difference increased with storage temperature: at 40 °C, T_25_% for KIO_3_ ranged from 87 to 98 days, while KI degraded much more rapidly, with T_25_% values of 55–65 days.

Preparation methods exerted a measurable, though secondary, influence on storage stability. BWC consistently yielded the lowest T_25_% values and highest decay constants (k), indicating the fastest degradation, while BWU showed the slowest decay and the highest T_25_%. CO occupied an intermediate position. The hierarchy of method stability (BWU > CO > BWC) was consistent across iodine forms, though differences diminished at high storage temperatures.

Model fitting was satisfactory, with R^2^ values ranging from 0.72 to 0.94 and RMSE values indicating good agreement between observed and predicted iodine dynamics (Table A1). Statistical analysis of the data (one-way ANOVA with Tukey post hoc test) revealed significant differences (*p* < 0.05) between KI and KIO_3_ for all preparation methods and storage temperatures, confirming the superior stability of KIO_3_ (Table 2).

Overall, the results indicate that iodine stability in fortified cauliflower is jointly influenced by iodine form, heat-treatment method, and storage temperature—but temperature and iodine form exert the strongest effects. KIO_3_ provides superior protection against thermal and storage-related losses, while KI is markedly more sensitive to degradation, particularly at elevated temperatures. Preparation-related effects are meaningful at lower temperatures but become limited under accelerated degradation conditions (40 °C), where temperature predominates.

### 3.2. Associations Between Iodine Content and Selected Phenolic Compounds

Correlation analysis (Table 3) revealed a complex and heterogeneous pattern of associations between iodine content and the analysed phenolic compounds—progoitrin (PROG), glucobrassicin (GB), gluconapin (GN), sinigrin (SINIG), and indole-3-carbinol (IK). These associations varied markedly depending on storage duration (30, 60, and 90 days), storage temperature (4 °C, 21 °C, and 40 °C), and the chemical form of iodine (KI or KIO_3_). The observed relationships were not uniform across experimental conditions, with frequent changes in both the magnitude and direction of correlation coefficients, indicating the absence of a single, consistent linear trend.

After 30 days of storage, correlations involving PROG were predominantly strong and negative for both KI and KIO_3_ across all temperatures (r from −0.705 to −1.000, *p* ≤ 0.05). In contrast, GB and GN displayed weaker and more variable associations, including non-significant correlations at 21 °C and 40 °C for KI. SINIG and IK were generally positively correlated with iodine content at 4 °C and 21 °C, particularly for KIO_3_, where very strong positive correlations were observed (r > 0.98), whereas at 40 °C SINIG showed a negative association with KI.

After 60 days, the correlation structure changed substantially. PROG exhibited strong positive correlations with KI at 4 °C (r = 0.870, *p* ≤ 0.05) but strong negative correlations at higher temperatures for both iodine forms. GB remained consistently negatively correlated with iodine content regardless of temperature or iodine form. In contrast, GN showed divergent behaviour, with a very strong positive correlation with KIO_3_ at 21 °C (r = 0.998, *p* ≤ 0.05) but negative or non-significant associations under other conditions. SINIG and IK were characterised by consistently positive correlations with both iodine forms across all temperatures.

After 90 days of storage, strong negative correlations between iodine content and PROG were observed across all temperatures and iodine forms (r from −0.756 to −0.987, *p* ≤ 0.05). GB showed a very strong negative association with KI at 4 °C (r = −0.999), while its correlations with KIO_3_ were weaker or non-significant at higher temperatures. GN exhibited temperature- and form-dependent behaviour, with positive correlations for KIO_3_ at 4 °C and 21 °C but negative correlations for KI at 21 °C and 40 °C. SINIG and IK generally maintained strong positive correlations with iodine content, particularly at elevated temperatures.

Overall, the correlation coefficients demonstrated substantial variability across storage time, temperature, and iodine form, including frequent sign reversals and isolated extreme values. These results indicate that observed linear associations between iodine content and phenolic compounds in fortified cauliflower are strongly condition-dependent and should be interpreted with appropriate statistical caution. The observed correlations most likely reflect context-specific patterns of co-variation, rather than stable or causal interactions.

**Figure 2:**
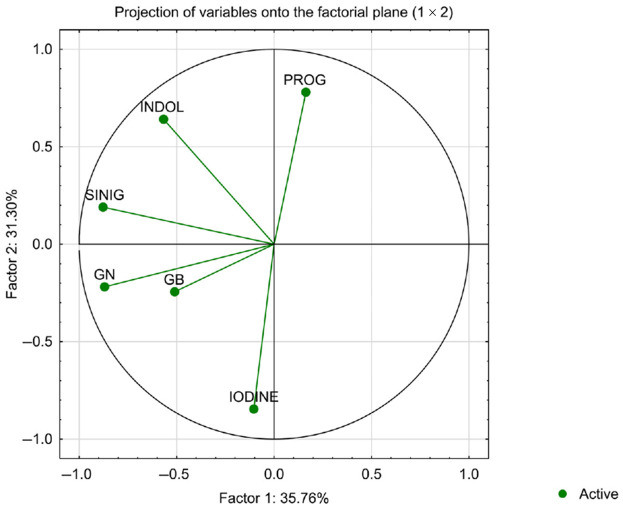
Principal component analysis (PCA) of the score plot of data from selected phenolic compounds (progoitrin [PROG], glucobrassicin [GB], gluconapin [GN], sinigrin [SINIG], and indole-3-carbinol [IK]) and iodine content in cauliflower.

The projection of the analysed variables onto the factorial plane (Figure 2) illustrates the relationships among the analysed variables projected onto the first two principal components, which together explain 66.86% of the total variance (Factor 1: 35.76%, Factor 2: 31.10%). The length and orientation of each vector indicate both the strength of its association with a given component and the correlations among variables. The relative proximity and orientation of vectors indicate similarities in variance structure rather than direct interactions.

Factor 1 is primarily defined by negative loadings of gluconapin (GN), glucobrassicin (GB) and sinigrin (SINIG), which cluster in the lower-left quadrant, suggesting that these glucosinolates share similar variance patterns and are strongly positively correlated with one another. Indole-3-carbinol (INDOL) also loads negatively on Factor 1 but is positioned higher on Factor 2, indicating partial association with this group while retaining an additional independent contribution to the second component.

Progoitrin (PROG) exhibits a strong positive loading on Factor 2 and minimal association with Factor 1, positioning it as the dominant descriptor of this axis. Its location suggests that progoitrin varies in a pattern largely distinct from the other glucosinolates.

Iodine loads negatively on Factor 2 and moderately on Factor 1, placing it opposite progoitrin along the vertical dimension. This distribution indicates that iodine levels are inversely associated with variations in progoitrin content, and only weakly correlated with the remaining metabolites.

Overall, the projection reveals two main gradients of variation: one related to the cluster of glucobrassicin, gluconapin, sinigrin and partially indole-3-carbinol (Factor 1), and another contrasting progoitrin with iodine (Factor 2). These patterns suggest that iodine retention and specific glucosinolate profiles respond differently to the experimental conditions, reflecting distinct underlying biochemical or processing-related mechanisms.

### 3.3. Antioxidant Activity

**Figure 3:**
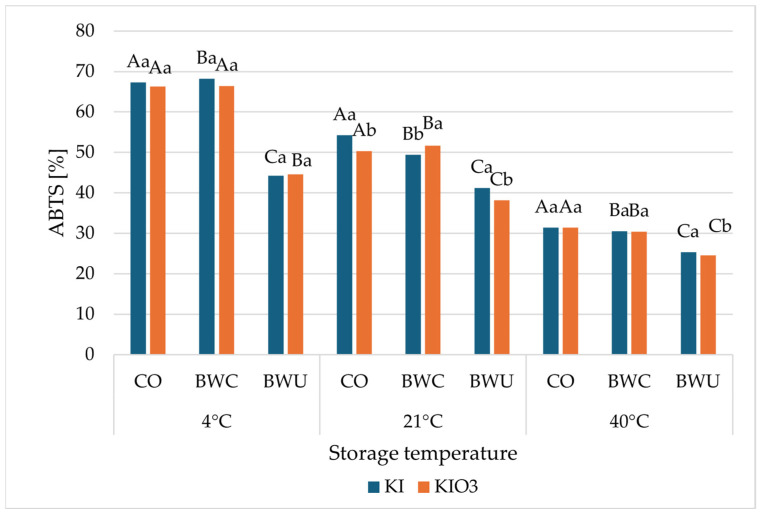
The ABTS^●+^-scavenging ability of cauliflower fortified with KI and KIO_3_ after 90 days of storage at different temperatures (4 °C, 21 °C, 40 °C) presented in relation to the applied heat-treatment methods (BWC—cooking in boiling water covered, BWU—cooking in boiling water uncovered, CO—steamed in a convection oven). Mean values (*n* = 6); different letters (lower letters in the same heat treatment method; upper case letters in the same form of iodine) denote a significant difference at *p* < 0.05 (one-way ANOVA and post hoc Tukey test). Statistical comparisons were conducted within groups of samples stored at the same temperature.

**Figure 4:**
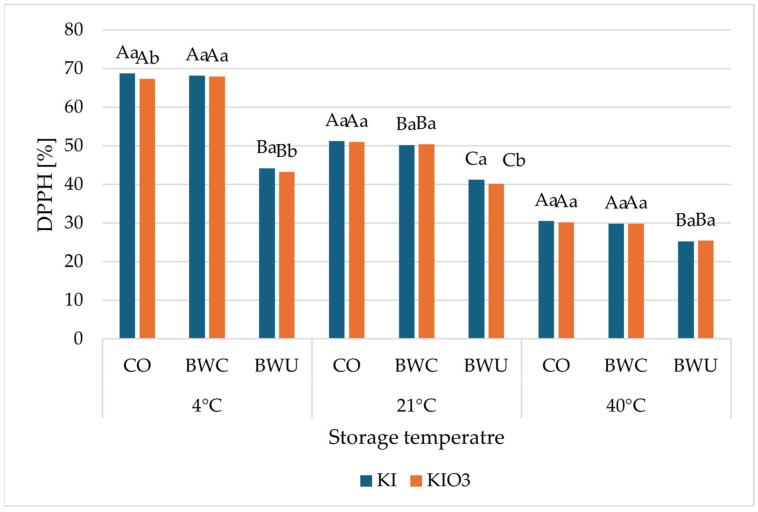
The DPPH^●^-scavenging ability of cauliflower fortified with KI and KIO_3_ after 90 days of storage at different temperatures (4 °C, 21 °C, 40 °C) presented in relation to the applied heat-treatment methods (BWC—cooking in boiling water covered, BWU—cooking in boiling water uncovered, CO—steamed in a convection oven). Mean values (*n* = 6); different letters (lower letters in the same heat treatment method; upper case letters in the same form of iodine) denote a significant difference at *p* < 0.05 (one-way ANOVA and post hoc Tukey test). Statistical comparisons were conducted within groups of samples stored at the same temperature.

The antioxidant activity of cauliflower fortified with potassium iodide (KI) and potassium iodate (KIO_3_) after 90 days of storage, expressed as ABTS^•+^- and DPPH^•^-scavenging capacity, is presented in relation to storage temperature and heat-treatment method (Figure 3 and Figure 4).

For both assays, a clear effect of storage temperature was observed. Regardless of the iodine form and heat-treatment method, samples stored at 4 °C exhibited the highest radical-scavenging activity, whereas storage at 40 °C resulted in the lowest values (Figure 3 and Figure 4). Intermediate values were generally recorded for samples stored at 21 °C.

In the ABTS^•+^ assay (Figure 3), cauliflowers subjected to steaming in a convection oven (CO) and cooking in boiling water, covered (BWC), exhibited higher ABTS^•+^-scavenging values than those treated with cooking in boiling water, uncovered (BWU) at all storage temperatures. This trend was consistent for both iodine forms. At 4 °C, ABTS^•+^-scavenging capacity ranged from approximately 66–68% for CO and BWC samples, while markedly lower values were observed for BWU-treated samples (44%). Increasing storage temperature led to a progressive decline in ABTS^•+^-scavenging ability across all treatments.

A similar pattern was observed for the DPPH^•^ assay (Figure 4). After 90 days of storage at 4 °C, the highest DPPH^•^-scavenging activity was recorded in CO- and BWC-treated samples, whereas BWU-treated cauliflowers exhibited substantially reduced values. Storage at 21 °C and 40 °C resulted in a pronounced decrease in DPPH^•^-scavenging capacity, particularly in samples subjected to BWU treatment.

When comparing iodine forms, cauliflowers fortified with KI generally exhibited slightly higher ABTS^•+^- and DPPH^•^-scavenging activities than those fortified with KIO_3_ across most heat-treatment methods and storage temperatures. However, the overall trends associated with storage temperature and heat treatment were consistent for both iodine forms.

## 4. Discussion

This study provides new insights into the stability of iodine in a vegetable matrix fortified post-harvest in relation to common culinary practices and storage conditions. The results demonstrate that both the method of thermal pre-treatment applied to the raw material prior to fortification and the storage temperature substantially affect iodine retention in fortified freeze-dried cauliflower. These observations are consistent with previous reports on iodine-enriched foods, which indicate that iodine losses are strongly dependent on temperature and the chemical form of iodine [17,38,48,49].

Notably, the relatively high iodine retention observed, particularly after long-term storage at 4 °C, indicates that cauliflower is a more stable carrier than table salt under comparable conditions, and performs similarly to previously examined protein or fibre-based matrices [41,47,50]. This supports the technological relevance of vegetables as alternative iodine carriers, in the context of WHO recommendations to reduce salt intake [7,8]. The main contribution of this study is to demonstrate how different thermal treatment methods applied to the raw material before fortification influence iodine retention in cauliflower after fortification and during subsequent storage. The results indicate that the applied heat pre-treatment prior to fortification contributes to differences in iodine stability. At the same time, the results confirm previous observations that the chemical form of iodine and storage temperature remain key determinants of iodine stability in vegetable matrices [38]. The particularly strong associations observed for KI at higher temperatures may reflect the greater susceptibility of iodide to oxidative degradation, whereas the more stable behaviour of KIO_3_ is consistent with its known resistance to thermal and oxidative loss [49,51,52]. Similar temperature-dependent patterns have been documented in other fortified vegetable matrices [38,53].

Differences in iodine retention between the applied heat-treatment methods suggest that thermal processing prior to fortification modifies matrix properties relevant to iodine stability. The lower iodine retention observed in samples boiled under cover, compared with uncovered boiling and steaming, may be associated with differences in the retention of volatile endogenous compounds typical of Brassica vegetables.

Limiting steam release may promote the accumulation of glucosinolate-derived volatiles and other aroma-active constituents within the matrix, which could influence iodine stability through redox or binding reactions. Conversely, boiling in water without cover or steaming likely promotes the release of such volatiles via continuous air circulation, resulting in higher iodine retention [23]. It should be emphasised, however, that the present study does not provide direct mechanistic evidence for these processes, and these observations should therefore be interpreted as descriptive effects of the applied processing conditions. They also highlight the need for targeted analytical studies to verify the underlying mechanisms.

At the same time, the study provides a preliminary assessment of whether changes in iodine retention coincide with changes in the content of selected goitrogenic phytochemicals, depending on the type of thermal treatment applied to the raw material before fortification. Correlation analysis revealed heterogeneous, condition-dependent associations between iodine content and individual phytochemicals, with frequent changes in both magnitude and direction across storage time, temperature, and iodine form.

These results suggest that variation in the content of the analysed phytochemicals may contribute to the observed variability in iodine retention. However, they do not support the existence of stable or universal linear relationships applicable to other Brassica vegetables. Accordingly, the observed associations should be regarded as exploratory indicators of context-specific co-variation rather than evidence of causal or mechanistic interactions.

From a technological perspective, understanding whether processing-induced changes in the phytochemical composition of the raw vegetable matrix—beginning already during preparation prior to fortification—influence iodine retention and stability is an important consideration for the rational design of iodine-fortified vegetable matrices. This is particularly relevant given the high antioxidant potential of many endogenous phytochemicals naturally present in Brassica vegetables.

Previous studies have demonstrated that the introduction of iodine compounds, which exhibit redox activity, into vegetable-based matrices may affect their antioxidant potential, as measured by ABTS^•+^ and DPPH^•^ assays, with statistically significant reductions in free radical scavenging capacity observed depending on both iodine concentration and chemical form. These findings suggest that iodine fortification may influence the redox properties of the matrix [19,20,53]. However, when considered from a matrix perspective, it may be hypothesised that processing-related changes in the content of naturally occurring antioxidant phytochemicals could influence the oxidative environment of the vegetable matrix and thereby co-occur with differences in iodine retention after fortification and during storage. However, this interpretation remains hypothetical and warrants further targeted investigation [38].

From a nutritional perspective, understanding these relationships is important because cruciferous vegetables are both an important source of health-promoting phytochemicals [54,55,56] and compounds with known goitrogenic potential [29,57], especially in populations that rely heavily on plant-based diets [13]. At present, it can only be hypothesised whether the introduction of iodine compounds into a vegetable matrix rich in goitrogenic constituents may reduce the anti-nutritional potential of the matrix or, conversely, hinder or even prevent iodine uptake from the fortified vegetable matrix, thereby limiting its usefulness in addressing iodine deficiency disorders (IDD). It may be possible that the selection of an appropriate fortification protocol, including suitable processing strategies and iodine forms, could help mitigate potential antagonistic effects while preserving both iodine stability and the nutritional value of the vegetable matrix. As iodine bioavailability was not assessed in the present study, these considerations remain speculative and should be addressed in future research.

Previous studies on post-harvest iodine-fortified vegetable matrices have further demonstrated not only high iodine retention immediately after fortification and satisfactory stability during storage under variable conditions [38], as well as their feasibility for incorporation into formulated foods. Although iodine-fortified freeze-dried vegetable products are not intended for direct household use—unlike table salt—they may serve as functional ingredients in food production. In this context, earlier work by Jankowska & Szymandera-Buszka (2024) [58] and Szymandera-Buszka et al. (2024) [59] showed that ciabatta rolls and gnocchi-type dumplings formulated with addition of iodine-fortified dried vegetables constituted effective dietary sources of iodine and may contribute to the prevention of iodine deficiency disorders (IDD) [58]. Importantly, these products were characterised by high sensory acceptability, and iodine fortification did not adversely affect their sensory profiles [59]. These findings indicate that iodine-fortified vegetable ingredients may represent a viable component of broader dietary strategies aimed at supporting iodine intake, while remaining aligned with public health recommendations to limit salt consumption.

This research fills a notable gap in the literature. In contrast to existing studies that have focused mainly on agronomic biofortification [60,61,62,63,64,65] the present research examined the stability of iodine in vegetables fortified post-harvest and the influence of pre-fortification thermal treatment of the raw material on iodine retention after fortification and during storage under typical conditions. By analysing these combined effects, the present study provides preliminary data and underscores the complexity of factors that must be considered when designing effective plant-based iodine matrices.

Several limitations of the present study should be acknowledged. First, the study was conducted using a single vegetable species and one fortification dose, which limits the generalisability of the findings to other plant matrices or fortification scenarios. Second, iodine bioavailability was not assessed, since the present work was intentionally focused on iodine retention, stability, and matrix-related effects rather than on physiological absorption or utilisation. Future studies incorporating in vitro digestion models and in vivo approaches would be required to address this aspect more comprehensively. Finally, while the correlation analysis suggests condition-dependent co-variation between iodine content and selected phenolic compounds, these relationships are exploratory in nature. The underlying mechanisms cannot be conclusively resolved without targeted metabolomic, kinetic, or mechanistic investigations, and the observed associations should therefore be interpreted with appropriate caution.

## 5. Conclusions

The effectiveness of using cauliflower as a matrix for iodine fortification was shown to depend on the applied preliminary thermal treatment. The cooking methods applied as a preliminary thermal treatment significantly influenced iodine retention after fortification and during storage, with boiling under cover proving to be the least favourable, whereas uncovered boiling and steaming resulted in higher iodine preservation. These findings indicate that the appropriate selection of preliminary thermal treatment is an important, but not exclusive, determinant of fortification efficiency in vegetable matrices.

The study further demonstrated that iodine stability in fortified cauliflower is associated with changes in the content of selected endogenous goitrogenic compounds as well as antioxidant activity. Correlation analysis revealed heterogeneous and condition-dependent relationships between iodine and selected phenolic goitrogenic compounds, varying with storage temperature and iodine form. In this context, iodine introduced as KI exhibited greater sensitivity to interactions with goitrogens than KIO_3_, highlighting the importance of selecting the chemical form of iodine in designing fortification procedures.

Storage conditions, particularly temperature, further affected iodine retention, with elevated temperatures accelerating iodine losses regardless of the preparation method. This confirms the necessity of considering storage conditions when assessing long-term iodine stability. Nevertheless, cauliflower generally demonstrated favourable properties as an iodine carrier, indicating its potential as a functional ingredient for populations at risk of iodine deficiency.

The study also confirmed the suitability of the applied fortification, drying, and storage protocols for assessing iodine stability in vegetable matrices. The results highlight the need for cautious interpretation of phytochemical–iodine relationships and indicate that further research is warranted to characterise the interactions between iodine, goitrogenic compounds, and processing parameters, as well as to extend these analyses to other vegetables that could serve as effective iodine carriers.

## Figures and Tables

**Table 1:** Iodine content [% **] in cauliflower enriched with KIO_3_ and KI depending on the heat treatment method (BWC—cooking in boiling water covered, BWU—cooking in boiling water uncovered, CO—steamed in a convection oven) and storage conditions (4 °C, 21 °C, 40 °C).

Iodine Form	Method of Preparation					
	CO		BWC		BWU	
	%	Standard Deviation	%	Standard Deviation	%	Standard Deviation
**After drying**						
4 °C						
KI	86.25 ^bC^*	0.16	91.25 ^bB^	0.22	93.05 ^bA^	0.11
KIO_3_	90.85 ^aC^	0.21	93.36 ^aB^	0.21	95.98 ^aA^	0.14
**After storage (90 days)**						
4 °C						
KI	82.92 ^bA^*	0.12	80.70 ^bB^	0.22	83.39 ^bA^	0.11
KIO_3_	84.32 ^aC^	0.11	86.02 ^aB^	0.21	87.92 ^aA^	0.14
21 °C						
KI	78.56 ^bA^*	0.11	77.06 ^bB^	0.20	78.61 ^bA^	0.11
KIO_3_	80.28 ^aC^	0.14	82.14 ^aB^	0.21	83.96 ^aA^	0.14
40 °C						
KI	70.49 ^bC^*	0.12	71.76 ^bB^	0.19	73.02 ^bA^	0.11
KIO_3_	74.04 ^aC^	0.13	75.76 ^aB^	0.21	76.82 ^aA^	0.14

* Mean values (*n* = 6); different letters (lower case letters in the same form of iodine; upper case letters in the same heat treatment method) denote a significant difference at *p* < 0.05 (one-way ANOVA, and post hoc Tukey test). ** Iodine retention was expressed as a percentage relative to the iodine content measured immediately after fortification.

**Table 2:** Statistical significance of predictors of variance models for changes in iodine content in selected iodine-fortified cauliflower after 90 days of storage (one-way ANOVA test).

Predictors	SS	df	MSE	F-Value	*p*-Value
Iodine form	67.10	1	67.10	2.93	0.11
KI					
Heat treatment	5.06	2	2.53	0.09	0.92
Storage temperature	170.03	2	85.01	57.44	0.00
KIO_3_					
Heat treatment	16.89	2	8.44	0.30	0.75
Storage temperature	170.24	2	85.12	29.69	0.00

SS—statistical significance; df—degrees of freedom; MSE—mean sum of squares.

**Table 3:** Correlation coefficients between the content of iodine and phenolic compounds (progoitrin (PROG), glucobrassicin (GB), gluconapin (GN), sinigrin (SINIG), and indole-3-carbinol (IK)).

Type of Phenolic Compounds	Iodine Form and Temperature of Storage					
	4 °C		21 °C		40 °C	
	KI	KIO_3_	KI	KIO_3_	KI	KIO_3_
After storage (30 days)						
PG	−0.796 ***	−1.000 ****	−0.705 ***	−0.978 ****	−0.726 ***	−0.972 ****
GB	−0.314 *	−0.874 ***	0.132 ^NS^	−0.561 **	−0.764 ***	−0.390 *
GN	0.434 **	−0.498 **	0.150 ^NS^	−0.070 ^NS^	0.079 ^NS^	−0.750 ***
SG	0.877 ***	0.998 ****	0.755 ***	0.633 **	−0.718 ***	0.791 ***
IK	0.644 **	0.990 ****	0.870 ***	0.978 ****	0.779 ***	0.922 ****
After storage (60 days)						
PG	0.870 ***	−0.827 ***	−0.949 ****	−0.954 ****	−0.969 ****	−0.896 ***
GB	−0.702 ***	−0.755 ***	−0.374 *	−0.481 **	−0.407 **	−0.845 ***
GN	−0.373 *	0.079 ^NS^	−0.722 ***	0.998 ****	−0.622 **	0.572 **
SG	0.912 ****	0.976 ****	0.755 ***	0.994 ****	0.990 ****	0.999 ****
IK	0.872 ***	0.917 ****	0.956 ****	0.908 ****	0.822 ***	0.995 ****
After storage (90 days)						
PG	−0.830 ***	−0.756 ***	−0.956 ****	−0.984 ****	−0.987 ****	−0.878 ***
GB	−0.999 ****	−0.439 **	−0.709 ***	0.080 ^NS^	−0.574 **	−0.400 **
GN	−0.168 ^NS^	0.826 ***	−0.603 **	0.822 ***	−0.809 ***	−0.739 **
SG	0.465 **	0.892 ***	0.996 ****	0.995 ****	0.953 ****	0.648 **
IK	0.990 ****	0.914 ****	0.904 ****	0.835 ***	0.890 ***	0.675 **

**** linear relationship very strong; *** linear relationship significant; ** linear relationship moderate; * linear dependence weak; NS no linear relationship; at: *p* ≤ 0.05; *n* = 14.

## Data Availability

The original contributions presented in the study are included in the article, further inquiries can be directed to the corresponding author.

## Abbreviations

The following abbreviations are used in this manuscript:

CO	Steamed in a convection oven
BWC	Cooking in boiling water covered
BWU	Cooking in boiling water uncovered
PROG	Progoitrin
GB	Glucobrassicin
GN	Gluconapin
SINIG	Sinigrin
IK	Indole-3-carbinol

## Appendix A

**Table A1:** The dynamic changes in iodine content during 90 days of storage of the dried iodine-fortified cauliflower were subjected to heat treatment in various methods (BWC—cooking in boiling water covered, BWU—cooking in boiling water un-covered, CO—steamed in a convection oven) and under different storage temperatures (4 °C, 21 °C, 40 °C).

Dynamic of Change in Iodine Content During 90 Days											
Temperature of Storage [°C]	Method of Preparation	T_25%_ [Days]	*R* ^2^	*RMSE*	k	A_0_ *	T_25%_ [Days]	*R* ^2^	*RMSE*	K *	A_0_ *
		KIO_3_					KI				
4 °C	CO	150.14	0.89	0.050	−0.004	11.38	96.84	0.86	0.076	−0.006	14.63
	BWC	174.07	0.94	0.073	−0.004	12.42	118.59	0.94	0.150	−0.005	10.42
	BWU	187.56	0.90	0.034	−0.004	15.93	119.75	0.88	0.045	−0.005	14.45
21 °C	CO	118.81	0.86	0.085	−0.005	11.12	76.36	0.79	0.114	−0.006	13.12
	BWC	131.78	0.87	0.135	−0.005	11.96	83.97	0.83	0.184	−0.006	13.78
	BWU	147.61	0.88	0.085	−0.005	15.29	91.79	0.82	0.124	−0.006	13.95
40 °C	CO	87.44	0.87	0.070	−0.007	10.88	55.06	0.72	0.114	−0.008	12.30
	BWC	92.06	0.86	0.293	−0.007	11.61	58.78	0.75	0.372	−0.008	12.90
	BWU	98.0	0.86	0.063	−0.007	14.74	64.90	0.79	0.096	−0.008	13.33

* A_0_—the initial amount of iodine, k—decay constant.
